# Long-Term Effects of Mind-Body Exercises on the Physical Fitness and Quality of Life of Individuals With Substance Use Disorder—A Randomized Trial

**DOI:** 10.3389/fpsyt.2020.528373

**Published:** 2020-12-18

**Authors:** Dong Zhu, Mei Jiang, Ding Xu, Wolfgang I. Schöllhorn

**Affiliations:** ^1^School of International Education, Shanghai University of Sport, Shanghai, China; ^2^Shanghai Drug Administration, Shanghai, China; ^3^Institute for Sport Science, Johannes Gutenberg-University Mainz, Mainz, Germany

**Keywords:** mind-body exercise, methamphetamine, heroin, tai chi, qi gong, yoga, rehabilitation, quality of life

## Abstract

**Background:** Mind-body exercises (MBE) are sequences of low to medium-intensity activities that benefit healthy performers physically and mentally. In contrast to the unmodified application of traditional tai chi, qi gong, or yoga in the healthy population, MBEs are typically tailored for individuals with substance abuse disorder (SUD). Despite numerous applications in practice, the detailed effects of tailor-made MBEs for SUD are unclear.

**Objectives:** This study aimed to analyze and compare changes in the physical fitness and quality of life of individuals with SUD that underwent conventional or tailor-made MBEs.

**Methods:** A total of 100 subjects obtained from the Shanghai Mandatory Detoxification and Rehabilitation Center with SUD were randomly assigned into two groups. The subjects in the experimental group (*n* = 50) practiced tailored MBE for 60 min a day, five times a week, for 3 months. The subjects (*n* = 50) in the control group were treated with conventional rehabilitation exercises with the same intervention protocol. The outcomes of fitness and quality of life for drug addiction were measured at the beginning and after 3 and 6 months by a questionnaire (QOL-DA). A two-way repeated measure analysis of variance was applied to compare the difference of treatments in the two groups.

**Results:** Statistically significant differences for the experimental group were found in systolic (*p* < 0.01, η^2^ = 0.124) and diastolic blood pressure (*p* < 0.01, η^2^ = 0.097), pulse (*p* < 0.01, η^2^ = 0.086), vital capacity (*p* < 0.05, η^2^ = 0.036), flexibility (*p* < 0.01, η^2^ = 0.143), and aerobic endurance (*p* < 0.01, η^2^ = 0.165). Results of the QOL-DA showed statistically significant differences between the experimental and control groups in total score (*p* < 0.01, η^2^ = 0.158) with greater effects on the former.

**Conclusions:** This study provided evidence that tailored MBE could lead to remarkable effects with regard to blood pressure, vital capacity, flexibility, and aerobic endurance in comparison with conventional rehabilitation methods.

**Clinical Trial Registration:** ChiCTR-IPR-14005343.

## Introduction

According to the 2019 World Drug Report, released by the United Nations Office on Drugs and Crime in June 2019, a total of 271 million people worldwide used illicit drugs in the previous year, whereby 35 million people were estimated to be suffering from substance use disorder (SUD). For people with SUD, the availability of and access to treatment services remain limited on the global level, as only one in seven people with SUD receives treatment each year (1). In China, 2.4 million drug users were registered nationwide, including 889,000 opioid abusers and 1,350,000 methamphetamine (MA) abusers, accounting for 42.1 and 57.3%, respectively, by the end of 2018 (2). SUD and related psychopathology are persistent problems in public health, which adversely affect the health and social functioning of sufferers, and are a major burden to their families and wider society (1–3). MA use is associated with an increased severity of cardiomyopathy, mental disorder, and damage to the brain caused by neurotoxicity (4–6). Chronic MA addicts exhibit impairment of cognitive functions and poor psychological well-being. Therefore, cognitive function must be considered as an important component in the treatment of MA dependence (7). The high relapse rate has been affecting the sustainability of anti-drug work worldwide. According to studies in China, the relapse rate for heroin abusers within the first year was 96.68% (8). To date, non-effective treatment methods are only available amongst amphetamine users (9).

Physical exercise (PE) within a conventional rehabilitation program is widely regarded as a form of “medicine” for the prevention and treatment of myriad somatic health conditions (10). Considerable evidence shows that sports can play a positive role in reducing illicit drug use (11). SUD is currently considered as a brain disease, and PE must be considered as an alternative treatment approach that can induce neuroplasticity on individuals with SUD (12). PE increases the concentration of neurotransmitters, which contribute to exercise-induced reward. In addition, a meta-analysis shows that PE increases the release of brain-derived neurotrophic factors compared with control conditions (13). PE also evokes hippocampal neurogenesis (14), a process that may positively affect stress-related disorders (10).

### Mind-Body Exercise (MBE) as a Potential Therapy for Chronic Diseases

MBE is often implemented in patients because of its low physically and emotionally expected risk, relatively low costs, and potential to allow patients to take a more active role in treatment (15). According to the US National Institutes of Health, National Center for Complementary and Integrative Health, mind-body practices focus on the interactions amongst the brain, mind, body, and behavior, with the intent to use the mind to affect physical functioning and promote health (16). Several mind-body therapies have been reported to decrease indicators of sympathetic activation (17–19). Mind-body approaches may satisfy unmet medical needs by relieving symptoms associated with chronic diseases in the management of mild-moderate mental and physical symptoms (15). MBE, such as yoga, tai chi or qi gong are known to have a sustaining influence on the behavioral, psychological, and physiological levels of observation. By performing slow, coordinated movements in harmony with specific breathing rhythms and techniques, MBE not only affects the memory, but also demands multiple high-order cognitions (e.g., perceptual speed, visual-spatial ability, attention, multitasking, and planning) to maintain their postural stability (20). A study on tai chi has provided evidence for its comparable effects on cognitive function and emotion in comparison with swimming, running, or square dancing (21). This impact is supported by studies that associate functional changes with neurophysiological mechanisms, such as increased brain-derived neurotrophic factor levels (22). Studies that connect plasticity in brain morphology and function, including processes central to executive function, which influence attention and memory, provide additional evidence of the far-reaching effects of MBEs (15, 17, 23, 24).

### Positive Effects of MBE on SUD

According to a number of studies, different MBEs are reported to have positive impacts on some aspects that are severely influenced by SUD (25, 26). Qi gong meditation contributes positively to addiction treatment outcomes, and meditative therapy may be more attractive for female drug abusers than for males (27). In a study report, heroin users that were treated with qi gong seemed to have an accelerated detoxification, increased immune function, and improved oxygen metabolism, which were beneficial if they persisted in qi gong practice (28).

As a form of MBE, yoga is also used as a therapy of SUD and is reported to have certain positive effects (29). A 12-weeks yoga intervention can reduce substance intake, relieve the stress of SUD, and support individuals with SUD in getting back to normal life (30). In addition, considerable studies have demonstrated the positive effects of yoga on patients with SUD of opium, heroin, alcohol, and tobacco, including the improvement of the body-mind environment, depressive symptoms, emotion status, and decreased craving (31–34). Notably, most results of these studies indicate the amelioration of life quality after yoga exercise.

Mindfulness meditation may be another important approach to treat addiction (35). A review showed that after mindfulness meditation training, individuals' substance craving would decrease (36), and after 8 weeks of training, the risk of relapse would significantly reduce (37). This training approach improves the executive functions in adolescents with MA use disorders (38). Emerging evidence has shown that mindfulness meditation can treat and prevent SUD and other behavioral disorders by increasing the activity in areas of the brain involved in mood regulation (39).

Compared with other MBE, tai chi emphasizes the coordination of the body, breathing, and mind, and as a potentially effective exercise method to improve brain health and slow down brain aging, tai chi is attracting increasing attention (40). The effectiveness of tai chi in improving cognitive functions is reported in previous studies (41). Meanwhile, several studies suggest its affirmative impact on the body and mind of patients suffering from SUD, including enhanced inhibitory control and reduced craving (42, 43). A 4-years follow-up study reported that only 9.5% of the individuals using amphetamine in the tai chi intervention group relapsed after intervention and 26.3% in the normal treatment group (44). Another study showed that tai chi could improve the quality of life and balance of amphetamine users (45).

Tai chi is a safe and acceptable exercise for individuals suffering from SUD. However, individuals with SUD in Shanghai Mandatory Detoxification and Rehabilitation Centers (SMDRC) often complain about the difficulty of learning and practicing tai chi sequences, particularly for those who entered SMDRC <3 months earlier. The Shanghai Drug Administration organizes a team to design an MBE and provide a safe and effective exercise for the new patients in SMDRC, which can be beneficial for individuals with SUD, physically and mentally. The MBE, also known as healthy mind exercise, is composed of tai chi, qi gong, and yoga. The criteria for the MBE is that it must be easy to learn, with low to moderate physical intensity. It is tailor-made for individuals with SUD in SMDRC who are sedentary with poor physical and cognitive functions. MBEs aim to improve participants' fitness, enhance the function of the motor and cardiovascular systems, and help participants to develop a satisfactory living habit and attitude after the intervention. However, related references that compare MBE with conventional treatment for the rehabilitation or treatment of individuals with SUD are limited, and the effects are unclear. Therefore, this experiment aimed to compare the physical fitness and mental effects of MBE intervention on individuals with SUD with that of the conventional exercise (CE) treatment in SMDRC.

## Materials and Methods

### Study Design

This study was a single-blind (assessor-blind), 6-months randomized control trial. Participants were individuals with SUD randomly allocated to the MBE or CE group. Written informed consent was received from all participants, and the trial was approved by the Institutional Review Board of Shanghai University of Sport in accordance with the Declaration of Helsinki.

### Setting

The trial was conducted in SMDRC, and participants of both groups received the same amount of intervention sessions that were organized on a basketball field under fair weather or in an indoor hall during rainy weather. Two well-trained Chinese traditional sport masters instructed the MBE in the MBE group, and experienced instructors from SMDRC provided conventional rehabilitation methods to the CE group. Experienced researchers conducted the assessment of fitness function and quality of life at baseline and after 3 and 6 months.

### Participants

A total of 100 male individuals with SUD voluntarily participated in the trial, and they were randomly assigned to the MBE group (*n* = 50) and CE group (*n* = 50). At the time of recruitment, these individuals received drug withdrawal treatment at SMDRC. The inclusion criteria comprised voluntary individuals who were (1) aged 18 years or above, (2) reported to be amphetamine users, and (3) had no severe medical conditions that would preclude their participation in physical activities. The exclusion criteria consisted of (1) diagnosis of Axis I psychiatric disorders in addition to SUD, (2) medical or neurological illnesses or trauma that affected the central nervous system, and (3) undergoing pharmacological treatment with psychotropic medications.

The participants in the MBE group had an average age of 32 ± 5 years, height of 172.7 ± 5.6 cm, weight of 74.5 ± 11 kg, and years of drug abuse of 9.42 ± 5.29. In addition, the participants in the CE group had an average age of 30 ± 5 years, height of 174.0 ± 6.0 cm, weight of 75.4 ± 17.3 kg, and years of drug abuse of 9.00 ± 5.41 years. No significant difference in age, height, and weight was observed between the two groups (*p* > 0.05) at the beginning of the experiment.

### Outcome Measures

Outcome measures were obtained to verify the changes in physical fitness and mental health amongst the participants as a result of the interventions. Fitness with health-related and skill-related components involved day-to-day activities. Health-related fitness included aerobic fitness, muscular fitness, flexibility, and body composition (46). According to the Manual and Standard of Chinese National Physical Fitness Evaluation (MSCNPFE), physical fitness included body shape, physiological function, and fitness tests (47). In this study, the physical fitness test strictly followed the instruction of MSCNPFE. Mental health was defined by WHO as a state of well-being, in which an individual could realize his or her own abilities, cope with the everyday tasks and normal stresses of life, work productively, and make a contribution to the community (48). The mental health test was performed using the questionnaire of quality of life for addicted abusers (QOL-DA).

#### Primary Outcome: Fitness Evaluation

Measurements were performed every morning at the same time. Blood pressure (BP) was measured under standardized conditions prior to other tests: participants were asked to rest for 5 min and to stop taking caffeine or tobacco products within the preceding 30 min. Body composition and body mass indexes (BMI) were measured with Omron HBF-305. A balance test was performed standing on one leg with eyes closed. A sport watch was used to record the duration of the balance test. A progressive aerobic cardiovascular endurance run (PACER) was carried out to measure the aerobic capacity of subjects following standardized procedures. The participants ran from one marker to another marker set 20 m apart whilst keeping pace with a pre-recorded cadence. The cadence was set to music and increased every minute. Participants were instructed to keep up with the cadence for as long as possible. The test was terminated when a participant failed to reach the appropriate marker in the allotted time two times or could no longer maintain the pace. The number of laps completed was recorded (49, 50). The heart rate was monitored during the PACER test with a heart-rate monitor system (ALA COACH, Tai Wan). Subjects were required to wear a heart-rate strap on the chest, and the heart rate of each subject was transmitted to ALA COACH after the test.

#### Secondary Outcome: QOL-DA

The QOL-DA questionnaire was developed by Chong-hua Wan in 1997 for drug-dependent patients in China. It consists of 40 items that measure four scales, including physiology (nine items; e.g., “do you feel lack of energy,” “do you have difficulty doing things”), psychology (nine items; “do you feel lonely,” “do you feel depressed”), society (11 items; “do you feel a lack of safety,” “are you adaptable to the environment”), and symptoms (11 items; “have you had diarrhea,” “have you had short breath”), and one independent item of self-evaluated health status (item 41). These items were rated on a five-point Likert scale. The scores from the four scales were calculated by the corresponding endorsed item scores that ranged from 9 to 45 (physiology and psychology) and from 11 to 55 (society and symptoms) (51, 52).

### Intervention

#### Experimental Group (MBE)

Based on the physical characteristics of individuals with SUD in SMDRC, movements were selected from qi gong, tai chi, and yoga. The intensity, difficulty, and duration of exercises and practice space were considered. Each section of MBE included acupoint massage of traditional Chinese medicine to dredge meridians, stretch the body, and relieve muscle tension. Movements were in harmony with breathing, and practicing this MBE involved both physical function and mental elements. The form was composed of nine movements with four repetitions for each movement. The whole form was about 10 min. Two repetitions were performed in each exercise session. The exercise was practiced three times a day, once in the morning, afternoon, and evening. The exercise intensity was 3.5 METs, and the average heart rate was 100 bpm measured on four healthy college students with K4B2 and Polar Team Pro heart-rate monitors prior to this study, which corresponded to a low to moderate intensity.

#### Control Group (CE)

The control group was assigned to participate in recreational activities, which included 5 min of recreational activities (the ninth Guang Bo Ti Cao), 5 min of gesture language exercises, and self-study as recommended by the Shanghai Drug Administration. The duration of the intervention in the control group was similar to that of the MBE. The participants in the CE group had the same exercise frequency, intensity, duration, and exercise time as those in the MBE group.

### Randomization

For this study, a total of 100 male illicit drug abusers were selected from SMDRC, coded as 01, 02, 03, …. The participants were randomly divided into two groups: 50 in the experimental group and 50 in the control group. The randomized numbers were generated using EXCEL for treatment observation.

### Statistics

A Pearson chi-squared test was applied for categorical variables of demography, and an independent sample *t*-test was applied for continuous variables at the baseline comparison of QOL-DA and fitness. The Pearson chi-squared test and independent sample *t*-test were used to compare the demographic and clinical characteristic differences of the two groups at baseline.

A two-way repeated measure analysis of variance (ANOVA) was performed to test whether the treatments were different after 3 and 6 months. Time (baseline, 3 and 6 months) was the within-group factor; groups (MBE and CE) were the between-group factors, and year of drug dependence was the covariate. A *post-hoc* test with Bonferroni correction was used to identify the group that showed difference when the ANOVA showed a significant interaction. Effect size was used with the partial eta square as η^2^. Statistical analyses were performed using SPSS 19.0 (Chicago, USA).

Data were reported as the mean values (plus SD), and the significance level was set to *p* < 0.05. The interpretation of the eta square followed the rule of thumb by Cohen (53) with >0.01 (small), >0.06 (medium), and >0.14 (large).

## Results

The demographic data showed no statistically significant difference between the MBE and CE groups with regard to educational level, occupation, marital status, type of drug used, and duration of substance dependence (*p* > 0.05). In this study, the age of participants was between 20 and 40 years, and most of them were young. In addition, most of the participants were bachelors or divorced. The majority of the participants were unemployed. In both groups, 70–80% of the subjects had been users of MA (Table 1).

**Table 1:** Demography of individuals with substance use disorder (*N* = 100).

**Contents**		**Mind-body exercise** **(*****N*** **=** **50)**		**Conventional exercise** **(*****N*** **=** **50)**		**P**
		***N***	**Percent (%)**	***N***	**Percent (%)**	
Age	20–30 years	20	40	27	54	0.88
	31–40 years	29	58	22	44	
	41–50 years	1	2	1	2	
Marital status	Married	16	32	11	22	0.96
	Single	25	50	29	58	
	Divorced	9	18	10	20	
	Widowed	0	0	0	0	
Education	Elementary	8	16	6	12	0.19
	Middle school	26	52	25	50	
	High school or equivalent	9	18	12	24	
	Junior college	7	14	6	12	
	College level or higher	0	0	1	2	
Occupation	Unemployed	26	52	21	42	0.67
	Owner	7	14	10	20	
	Service	0	0	1	2	
	Farmer	1	2	0	0	
	Worker	5	10	1	2	
	Driver	4	8	1	2	
	Staff	4	8	7	14	
	Others	3	6	8	16	
Type of drug	Heroin	16	32	13	26	1.25
	Methamphetamine	37	74	41	82	
	Ketamine	5	10	2	4	
	Cocaine	1	2	0	0	
	Ecstasy	4	8	2	4	
	Marijuana	2	4	2	4	
	Times of treatments	1.94 ± 1.50		1.48 ± 0.86		0.56
	Years of drug dependents (years)	9.42 ± 5.29		9.00 ± 5.41		0.49

**Figure 1:**
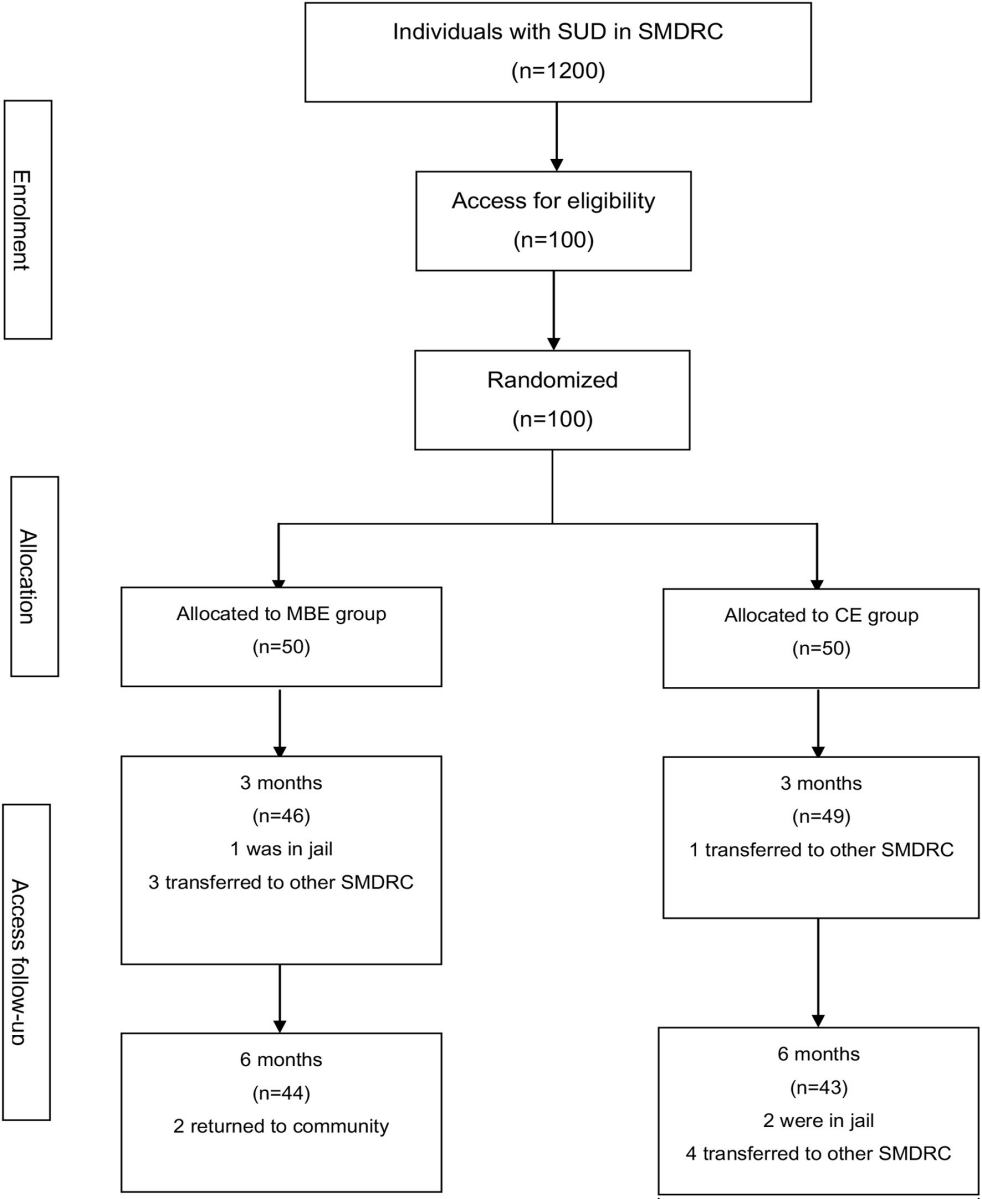
Flow diagram of the intervention progress through the phases of the two groups.

Six subjects in the MBE group and six subjects in the CE group dropped out during the intervention (Figure 1).

At baseline, no statistically significant differences were observed between the groups with regard to the physiological and physical fitness outcomes and scores of QOL-DA. The systolic pressure (SBP) in both groups was considered as elevated BP because the SBP was over 120 mmHg (Table 2).

**Table 2:** Physiological and physical fitness outcome comparison between the MBE and CE groups at the baseline.

**Items**	**Mind-body exercise** **(*n* = 50)**	**Conventional exercise** **(*n* = 50)**	***T***	***P*-value**
BMI (kg/m^2^)	24.91 ± 3.26	25.52 ± 3.89	−0.860	0.392
Body fat (%)	23.41 ± 4.56	23.37 ± 4.34	−0.043	0.966
Systolic (mmHg)	128.19 ± 13.88	129.54 ± 17.51	−0.424	0.673
Diastolic (mmHg)	79.67 ± 12.44	76.16 ± 11.48	1.458	0.148
Pulse (bpm)	75.58 ± 14.57	73.65 ± 12.20	0.712	0.478
Vital capacity (ml)	3059.20 ± 721.19	3128.78 ± 841.44	−0.442	0.659
Fundamental metabolize (kcal)	1674.20 ± 163.18	1723.63 ± 202.21	−1.340	0.183
Sit-and-reach (cm)	5.09 ± 8.07	7.28 ± 7.75	−1.379	0.171
One-leg stand with eyes closed (s)	30.38 ± 24.03	34.22 ± 29.47	−0.707	0.481

### Physiological Outcomes

**Figure 2:**
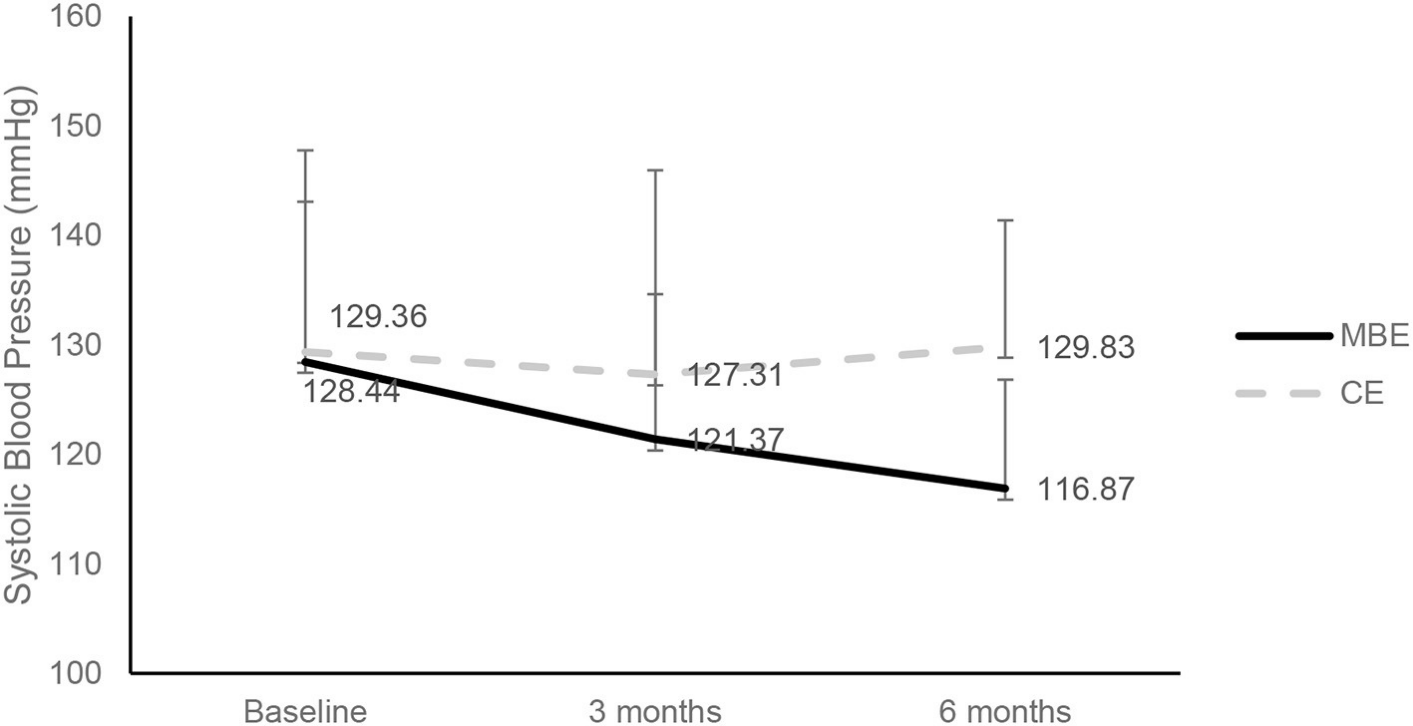
Comparison of systolic blood pressure between the two groups at the different phases (unit: mmHg). MBE, mind-body exercise group; CE, conventional exercise group.

Statistically significant differences were found in the time × group interaction after 6 months regarding BMI [*F*_(2,166)_ = 3.99, *p* < 0.05, η^2^ = 0.046], systolic blood pressure (SBP) [*F*_(2,166)_ = 11.77, *p* < 0.01, η^2^ = 0.124], diastolic blood pressure (DBP) [*F*_(2,166)_ = 8.96, *p* < 0.01, η^2^ = 0.097], pulse [*F*_(2,166)_ = 7.82, *p* < 0.01, η^2^ = 0.086], and vital capacity [*F*_(2,166)_ = 3.08, *p* < 0.05, η^2^ = 0.036]. The *post-hoc* analyses further revealed a significantly lower mean ± Std of SBP in the MBE group than that in the CE group (122.27 ± 1.98 vs. 128.78 ± 2.02 mmHg; *p* = 0.024). However, the vital capacity was significantly lower in the MBE group than that in the CE group (3006.55 ± 74.33 vs. 3222.64 ± 76.09 ml) after 6 months (Figure 2).

### Physical Fitness Outcomes

Statistically significant differences were found in the time × group interaction after 6 months with regard to sit-and-reach [*F*_(2,166)_ = 13.85, *p* < 0.01, η^2^ = 0.143], PACER [*F*_(2,166)_ = 15.05, *p* < 0.01, η^2^ = 0.165], and heart rate during the PACER test [*F*_(2,166)_ = 35.51, *p* < 0.01, η^2^ = 0.328]. *Post-hoc* analyses showed that the MBE group had a significantly higher mean ± Std laps of PACER (12.89 ± 0.42 vs. 11.47 ± 0.47; *p* = 0.029) and a significantly lower heart rate (105.38 ± 1.65 vs. 119.30 ± 1.78 bpm; *p* < 0.001) than the CE group during the PACER test.

### Mental Outcomes

Statistically significant differences were also observed between the two groups with regard to QOL-DA scores: physiology [*F*_(2,172)_ = 10.47, *p* < 0.01, η^2^ = 0.11], psychology [*F*_(2,172)_ = 9.82, *p* < 0.01, η^2^ = 0.102], symptom [*F*_(2,172)_ = 6.50, *p* < 0.01, η^2^ = 0.07], and society [*F*_(2,172)_ = 15.08, *p* < 0.01, η^2^ = 0.149], and the total score of QOL-DA [*F*_(2,172)_ = 16.13, *p* < 0.01, η^2^ = 0.158] was increased in the MBE group, whereas the scores in the CE group decreased after 6 months (Table 3).

**Table 3:** Comparison of the two groups at baseline, 3 and 6 months by ANOVA repeat measures.

	**MBE (*****N*** **=** **44)**			**CE (*****N*** **=** **43)**			**Time × Group (F)**	**Eta**
	**Baseline**	**3 Months**	**6 Months**	**Baseline**	**3 Months**	**6 Months**		
**MENTAL OUTCOMES**								
Physiology (PH)	32.68 (6.83)	34.20 (5.63)	34.86 (6.67)	33.20 (6.54)	32.66 (7.03)	27.41 (8.81)	10.47^**^	0.11
Psychology (PS)	32.36 (8.50)	35.86 (6.60)	36.25 (7.79)	33.77 (7.38)	33.80 (8.43)	27.73 (10.44)	9.82^**^	0.102
Symptom (ST)	47.86 (6.82)	48.27 (7.10)	48.23 (6.33)	47.70 (6.91)	48.39 (7.18)	42.43 (8.37)	6.50^**^	0.07
Society (SO)	38.25 (6.34)	38.77 (7.77)	41.73 (6.06)	39.45 (6.84)	39.89 (6.69)	34.61 (7.94)	15.08^**^	0.149
Total score	151.16 (23.29)	157.11 (20.57)	161.07 (21.23)	154.14 (24.03)	154.73 (24.95)	132.18 (30.76)	16.13^**^	0.158
**PHYSIOLOGICAL OUTCOMES**								
Mass (kg)	74.74 (11.46)	72.70 (11.78)	71.09 (11.73)	75.47 (17.94)	75.54 (13.75)	75.11 (12.93)	2.39	0.028
BMI (kg/m^2^)	25.10 (3.37)	24.40 (3.42)	23.61 (3.46)	25.69 (3.95)	25.03 (4.00)	24.80 (3.86)	3.99^*^	0.046
Body fat (%)	23.85 (4.59)	21.71 (4.96)	20.62 (5.09)	23.66 (4.11)	22.08 (4.65)	21.42 (4.85)	2.84	0.033
Systolic (mmHg)	128.44 (14.64)	121.37 (13.24)	116.87 (9.97)	129.36 (18.39)	127.31 (18.62)	129.83 (11.55)	11.77^**^	0.124
Diastolic (mmHg)	79.87 (13.03)	75.54 (9.46)	74.67 (8.41)	75.98 (12.14)	76.06 (11.57)	78.83 (10.91)	8.96^**^	0.097
Pulse (bpm)	75.02 (14.82)	70.60 (12.23)	69.44 (7.23)	73.02 (12.10)	65.34 (8.71)	72.52 (10.64)	7.82^**^	0.086
Basic metabolism (kcal)	1675.39 (168.48)	1655.95 (173.86)	1632.77 (172.18)	1728.71 (204.87)	1699.29 (203.74)	1686.17 (197.99)	0.82	0.01
Vital capacity (ml)	2994.75 (659.25)	2870.41 (632.59)	3150.89 (526.52)	3216.12 (811.43)	3275.05 (547.24)	3180.52 (597.89)	3.08^*^	0.036
**PHYSICAL FITNESS OUTCOMES**								
Sit-and-reach (cm)	4.64 (8.38)	6.20 (7.68)	11.82 (6.77)	7.34 (7.65)	8.21 (7.50)	8.23 (8.08)	13.85^**^	0.143
One-leg stand with eye closed(s)	30.55 (25.35)	30.56 (26.49)	36.11 (20.77)	30.41 (21.70)	26.70 (15.52)	27.64 (14.99)	1.71	0.02
PACER (laps)	16.05 (5.39)	14.23 (4.32)	8.37 (3.51)	18.19 (3.70)	11.83 (4.73)	4.39 (1.44)	15.05^**^	0.165
Running hear rate (bpm)	109.72 (17.01)	104.21 (12.01)	102.09 (11.99)	107.70 (13.13)	123.41 (15.27)	126.95 (11.86)	35.51^**^	0.328

MBE, mind-body exercise; CE, conventional exercises; data presented as mean (SD);

* p < 0.05;

** *p < 0.01*.

## Discussion

In this study, we compared the physical and mental effects of an MBE intervention with the conventional rehabilitation approach. The participants in the MBE group had statistically better physical function and mental outcomes than those in the CE group. Moreover, the MBE group showed better effects on BMI, SBP, pulse, and PACER test. The findings indicated that the MBE intervention might be beneficial for weight control and hypertension.

### Physical Fitness Benefit From the MBE

#### Relationship Between Weight Gain and Aerobic Capacity

In general, weight gain in illicit drug abusers is interpreted as a positive treatment outcome of amphetamine (54) because one of the effects of MA is suppressing appetite (55, 56). Based on related research, average BMI was significantly lower amongst MA abusers compared with the age-matched healthy controls reported by a survey in China (57). Methadone treatment and MA compulsory treatment were associated with weight gain (45, 58). In particular, fat mass increased after a certain period of treatment once the effects of the illicit drugs on appetite suppression and metabolism were removed (59). The statistically significant weight loss measured by the BMI decreased in the MBE group, but no changes were found in the CE group, indicating that the MBE might be a gentle and potential exercise to control weight gain during the treatment of SUD. MBE was suitable for those who do not want to participate in procedures with vigorous exercises.

Tai chi, qi gong, and yoga were assumed to have beneficial effects on the cardio-respiratory function of adults at different ages (60–62). In our study, aerobic endurance gradually decreased in both groups. This result was consistent with our previous study where the laps of the PACER test decreased in the experimental and control groups (44). However, the PACER test in the MBE group gradually decreased compared with the CE group. The sedentary behavior of subjects in mandatory detoxification and rehabilitation centers (MDRC) might raise the risk of cardiovascular diseases, such as hypertension and psychological diseases, such as depression because of weight gain. Physical activities are recommended for these subjects because sedentary behavior increased the prevalence of getting into MDRC. The results showed that MBE could alleviate the deterioration of endurance capacity amongst individuals with SUD and protect against weight gain during rehabilitation in MDRC.

Most movements in MBE were composed of elements of qi gong. As reported in many studies, qi gong has a number of potential advantages for respiratory function promotion through the action of qi gong forms with diaphragmatic muscle activity for deep breathing. By making the human thorax fully widen and narrow, the respiratory muscles are fully trained whilst practicing these movements. In addition, the combination of chest and abdominal breathing increased the activity of the diaphragm and abdominal muscles, thereby regulating the intercostal muscles and respiratory function, causing an increase in pulmonary ventilation (63, 64). The positive effects of breathing on the regulation of emotions (65) and cognitive functions, particularly with lower frequency, as enforced by qi gong, were reported and seemed to play a role in mindfulness training (66, 67). Although the *post-hoc* analysis revealed that the vital capacity in the MBE group after 6 months was lower than that in the CE group, the outcome of vital capacity in the MBE group was found to increase slightly from 2,995 ml at baseline to 3,151 ml after 6 months, and the result in the CE group was decreased from 3,216 ml at baseline to 3,181 ml after 6 months.

#### BP and Pulse

Previous systematic reviews indicated that TC might be safe and effective in reducing BP, improving aerobic capacity, and exercising tolerance for patients with cardiovascular conditions and risk factors (68–70). In that study, the BP and heart rate of individuals with SUD decreased in the MBE group, whereas no significant changes in BP and heart rate were found in the CE group. The SBP in the MBE group decreased to 11.57 mmHg, whereas no changes were found in the CE group. A reduction of 5 mmHg might reduce the risk of hypertension-related diseases (71). The SBP and DBP changes in individuals with SUD were consistent with the findings from earlier studies (44, 45, 60). Studies found that reductions in SBP and DBP were observed in several trials of qi gong interventions (72). A related study reported that the qi gong group showed a statistically significant improvement in ventilation functions, whereas no change was found in the control group after 10 weeks of practice (73). Tai chi, qi gong, and yoga MBEs using meditation, imagery, breathing, general relaxation, and stress reduction played active roles in the overall effects whilst practicing (74). The reduction of BP might stem from the enhancement of the parasympathetic tone and reduction of the sympathetic tone during the MBEs, such as relaxation training (75). Based on related studies, the low BP levels after MBE were compatible with the stabilization of the parasympathetic nervous system (PNS) and sympathetic nervous system (SNS) activities because BP has been shown to be directly linked to PNS and SNS activities (73, 75, 76).

The results of the PACER test showed that the number of running laps in the MBE group and CE group continued to decrease. The number of running laps in the MBE group decreased from 16 laps at baseline to 8 laps after 6 months. On the contrary, the number of running laps in the CE group dropped from 18 laps at baseline to 4 laps after 6 months, showing a drop of 14 laps. *Post-hoc* analysis indicated that a highly significant difference could be observed between the PACER test results in the two groups. The PACER test results in both groups indicated that the aerobic endurance capacity of individuals with SUD continuously decreased, and it was hardly maintained amongst individuals with SUD in SMDRC. Therefore, more exercises that focused on improving the cardiovascular function were encouraged in MDRC.

The MBE group ran more laps than the CE group. In addition, the average heart rate in the MBE group was lower than that in the CE group after 6 months. The pulse rate test during the PACER test in the MBE group continued to decline from 110 bpm at baseline to 102 bpm and decreased by 8 bpm during the same time span. By contrast, the average heart rate in the CE group continued to increase over the duration of the experiment, from 108 bpm at baseline to 127 bpm, showing an increase of 19 bpm over 6 months. Combined with the decrease of BP, the results indicated that MBE could have a beneficial effect on the cardiovascular system.

### Mental Benefit From the MBE

The scores of QOL-DA with regard to physiology, psychology, symptom, and societal function were significantly improved in the MBE group over 6 months, whereas these outcomes decreased in the CE group over time. Our findings were consistent with those of the previous study where MBE, such as tai chi was beneficial to SUD regarding the quality of life. The physical function and physiological changes with regard to the results of the mental function improvement and the scores of psychology, symptom, and society were not significantly improved in the MBE group, and such results were poor in the CE group. Consequently, MBE might help individuals with SUD in improving their quality of life.

### Limitations and Recommendations for Future Research

This study has several limitations. Firstly, the results of PACER were strongly related to participant's momentary motivation and attitude. Consequently, in future studies, incentive tests should be conducted to motivate the subjects to try their best during PE tests, which required high tolerance. Secondly, moderate-intensity continuous training or high-intensity interval training for improving aerobic capacity was not arranged because of the limited number of individuals with SUD who voluntarily participated in this study. Finally, considering that the study only included male individuals with SUD, whether gender difference might influence the physical fitness and mental effects remained unclear.

Further studies might continuously focus on the effects of exercise interventions on weight control of individuals with SUD because of the high risk of cardiovascular diseases caused by weight gain. Weight gain was a positive effect of abstinence because appetite suppression caused by illicit drug use was diminished, but the potential cardiovascular diseases brought on by inactivity might deteriorate the health of individuals with SUD.

## Conclusion

Our findings indicate that MBE has better physical fitness and mental effects than the conventional physical rehabilitation method. This approach may help participants in maintaining their body weight, decreasing the deterioration of aerobic capacity, and alleviating the risk of cardiovascular diseases. The quality of life of individuals with SUD can be improved by continually practicing MBE after intervention.

## Data Availability Statement

The datasets generated for this study are available on request to the corresponding author.

## Ethics Statement

The studies involving human participants were reviewed and approved by Shanghai University of Sport. The patients/participants provided their written informed consent to participate in this study.

## Author Contributions

DZ designed this study, analyzed the data, and wrote the manuscript. MJ conducted the exercise intervention, collected the data, and organized the measurement. DX study coordinating, study design, and research project advised. WS involved in the part of discussion and revised the manuscript. All authors contributed to the article and approved the submitted version.

## Conflict of Interest

The authors declare that the research was conducted in the absence of any commercial or financial relationships that could be construed as a potential conflict of interest.
